# Mid-term comparative study between the glenoid and humerus lateralization designs for reverse total shoulder arthroplasty: which lateralization design is better?

**DOI:** 10.1186/s12891-023-06383-0

**Published:** 2023-04-14

**Authors:** Hwan-Hee Lee, Sang-Eun Park, Jong-Hun Ji, Hyun-Sik Jun

**Affiliations:** 1 Department of Orthopedic Surgery, The Catholic University of Korea, Seoul, Republic of Korea; 2 Department of Orthopedic Surgery, Daejeon St. Mary’s Hospital, The Catholic University of Korea, 64 Daeheung-Ro, Jung-Gu, Daejeon, 34943 Korea

**Keywords:** Reverse total shoulder arthroplasty, Lateralization design reverse total shoulder arthroplasty, Glenoid lateralization, Humeral lateralization, Complication

## Abstract

**Introduction:**

The complications of the conventional medialized design for reverse total shoulder arthroplasty (RSA) are increased scapular notching, and decreased external rotation and deltoid wrapping. Currently, lateralization design RSA, which avoid scapular notching and improve impingement-free range of motion, is commonly used. Especially, humeral lateralization design was most commonly used and glenoid lateralization design was preferred for glenoid abnormities. We compared mid-term clinical and radiologic outcomes of glenoid and humeral lateralization RSA in an Asian population in this study.

**Materials and Methods:**

We enrolled 124 shoulders of 122 consecutive patients (mean age 73.8 ± 6.8 years) who received glenoid or humeral lateralization RSA from May, 2012 to March, 2019. We divided these patients into two groups according to RSA using either glenoid or humeral lateralization design. These different designs were introduced consecutively in Korea. The clinical and radiological results of 60 glenoid lateralization RSA (Group I, 60 patients) and 64 humeral lateralization RSA (Group II, 62 patients) were retrospectively evaluated and also were compared between the two groups. All patients were followed for mean 3 years.

**Results:**

The clinical and radiologic outcomes of the two groups did not differ significantly, including scapular notching (*p* = 0.134). However, humeral lateralization RSA showed a larger glenoid-tuberosity (GT) distance (*p* = 0.000) and less distalization shoulder angle (DSA) (*p* = 0.035). The complication rate did not differ significantly either. But, revision surgery was performed for 2 humeral loosening in the Group II.

**Conclusion:**

The clinical and radiologic outcomes of the two groups did not differ significantly, including scapular notching at mid-term follow-up. However, humeral lateralization design showed larger GT distance and less DSA. Humeral lateralization design RSA could preserve the normal shoulder contour due to a larger GT distance (more lateralization) and provide less deltoid tension due to less DSA (less distalization of COR).

## Introduction

The basic biomechanical principal on which reverse total shoulder arthroplasty (RSA) works is the medialization of the center of rotation (COR) and the distalization of the humerus. However, follow-up results have shown that the original Grammont prosthesis (medialized glenoid and medialized humerus design RSA) is associated with high rates of complications such as instability, infection, and scapular notching, scapular impingement of the humeral component in adduction that becomes radiographically apparent as bone loss at the scapular neck [1–6]. Long term outcomes studies on this medialized design demonstrated increased scapular notching and decreased external rotation and deltoid wrapping; therefore, the conventional RSA design has been modified to minimize those complications and maximize impingement-free range of motion (ROM). There has been rapid development of different prostheses where substanstial changes have been made to improve the geometry of the reverse shoulder prosthesis. Compared to the conventional medialized RSA design, lateralization design (lateral glenoid or lateral humerus RSA designs) has been reported to be a viable option. Both the glenoid and humeral lateralization RSA designs avoid the pitfalls of the conventional RSA and improve impingement-free ROM [5, 7, 8]. The modified RSA involve lateralization of the COR, either with bony increased offset (BIO)-RSA [9–11] or metallic increased offset-RSA [12] (glenoid lateralization design) or with an implant with a lower neck-shaft angle (NSA) (humeral lateralization design) [13, 14]. Lateralization of the glenoid leads to increased internal and external rotation and has been reported to decrease the incidence of scapular notching [9]. Boileau et al. reported that the advantages of using an autograft harvested in situ (BIO-RSA) include bone stock augmentation, lateralization, low donor-site morbidity, low cost, and adequate flexibility to simultaneously correct posterior and superior glenoid defects [10, 11]. Both bony (BIO-RSA) and metallic (DJO Surgical, Austin, TX, USA) lateralization of the glenoid component are reported to be viable options for increasing impingement-free ROM [10, 15]. However, due to the increasing stress at the glenosphere/glenoid interface, early failure of the glenoid component could be considered in this design.

In the humeral lateralized design, decreasing the humeral NSA or providing a more anatomic humeral inclination offers a significant increase in impingement-free ROM except for abduction and also reduces scapular notching effectively [8, 16]. Compared to Grammont design, onlay curved-stem RSA (lateralized humeral design) is associated with low rates of scapular notching and glenoid radiolucency, and humeral bone remodeling with decreased humeral inclination has demonstrated improved adduction, extension, and external rotation [5]. In a comparative study of medialized versus lateralized design RSA, the lateralized glenoid design (DJO Surgical, Austin, TX, USA) RSA with an NSA of 135° achieved greater external rotation and more normal anatomy of the shoulder than the medialized glenoid design (Grammont design prosthesis with an NSA of 155°) [17]. However, both lateralization design might cause higher tension in the soft tissue and periarticular joint capsule than the traditional RSA and might also lead to difficult prosthesis reduction during surgery in Asian people of small stature. Another comparative cohort study of standard RSA (no glenoid lateralization design) and BIO-RSA (glenoid lateralization design) using a curved, short-stem (humeral lateralized design) showed that the humeral lateralization design alone was sufficient to decrease notching and improve external rotation [18]. Therefore, among Asians of small stature, either humeral lateralization or glenoid lateralization could decrease notching and improve external rotation.

Until now, few studies have compared the glenoid versus humeral lateralized RSA designs, and this study is the first to compare the clinical and radiological outcomes of the two different designs (glenoid lateralization design vs humeral lateralization design) in an Asian population. In our study, we used the glenoid and humeral lateralization designs consecutively because the humeral lateralized design was introduced in Korea later than the glenoid lateralized design.

The purpose of this study was to compare the clinical and radiographic outcomes of patients who received either glenoid or humeral lateralization RSA. We hypothesized that glenoid lateralization design show poorer clinical and radiological outcomes including glenoid loosening than humeral lateralization design at mid-term follow up.

## Materials and methods

### Study population and implant design

**Fig. 1:**
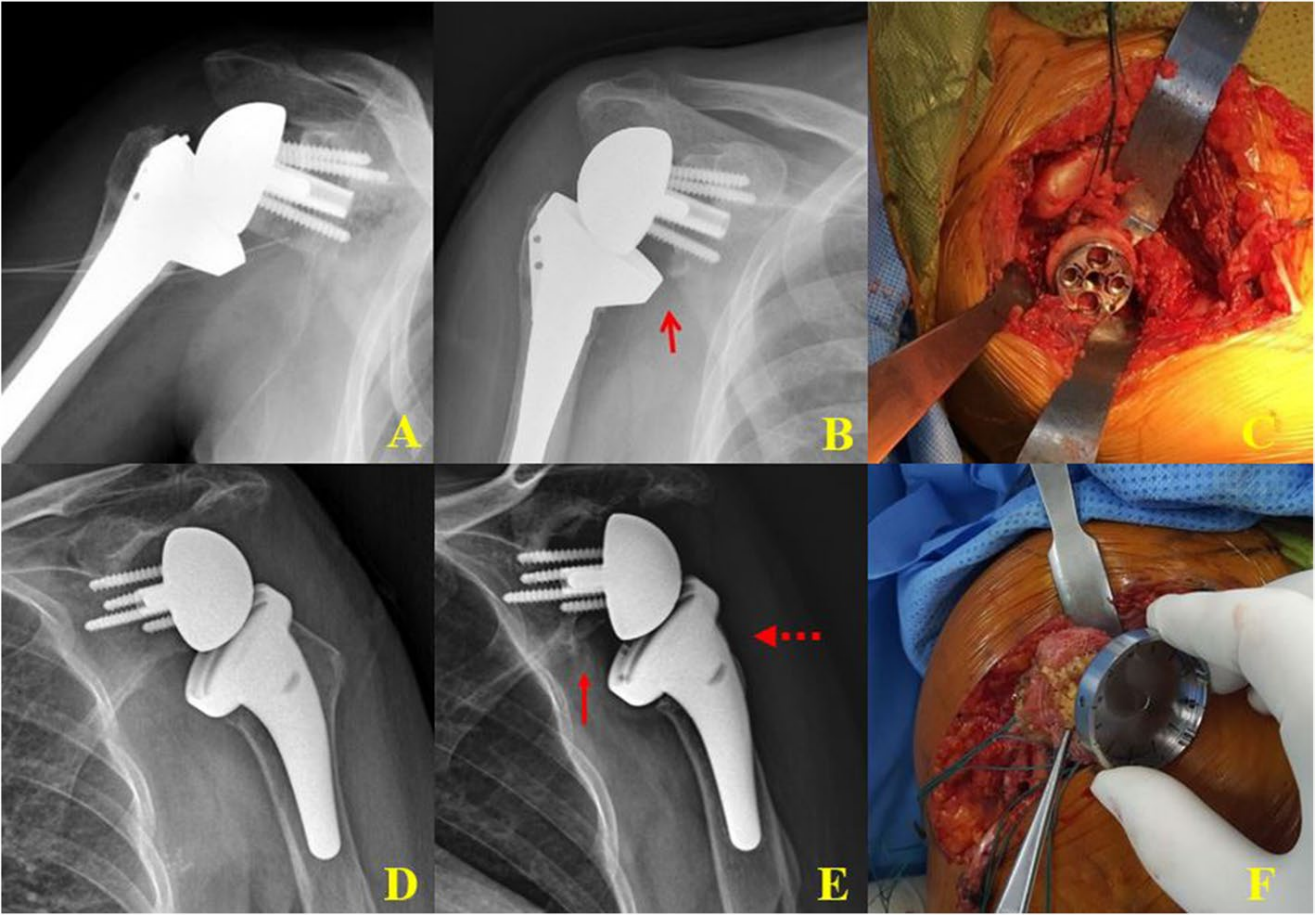
Comparison of glenoid lateralization (bony increased offset) reverse total shoulder arthroplasty) and humeral lateralized (cementless, curved, short-stem) reverse shoulder arthroplasty. Radiologic findings show the inserted bone graft on the glenoid in the glenoid lateralization design (**A**). A 4-year follow-up X-ray showing Grade 3 scapular notching on the scapular neck (**B**). Intraoperative photo showing that the autograft was well attached to the glenoid long post baseplate and inserted into the native glenoid (**C**). Radiologic findings showing the curved, short-stem insertion in the humeral lateralization design (**D**). A 3-year follow-up X-ray showing Grade 3 scapular notching on the scapular neck (**E**). Intraoperative photo showing that the chip bone autograft was inserted into the proximal humerus before stem insertion to prevent early humeral stem loosening (**F**)

This was a retrospective, single-center study of two consecutive case series using different RSA designs (glenoid and humeral lateralization) between May 2012 and March 2019 (Table 1). Initially, 138 patients were analyzed, and among these, 16 patients were excluded based on exclusion criteria. The inclusion criteria were patients who received either humeral or glenoid lateralized RSA to treat rotator cuff arthropathy or massive rotator cuff tears with pseudo-paralysis. The exclusion criteria were patients diagnosed with a proximal humeral fracture or inflammatory arthritis, revision surgery of a previous arthroplasty. Finally, a total of 124 shoulders of 122 consecutive patients (mean age 73.8 ± 6.8 years) were analyzed (Fig. 1). We divided these 124 shoulders into two groups: Group I (60 patients, 60 shoulders) received glenoid lateralization design RSA [Bony increased offset (BIO)-RSA]. Group II (62 patients, 64 shoulders) received humeral lateralization design RSA (Ascend Flex, cementless, curved short-stem RSA). Two patients received curved, short-stem RSA in both shoulders.

**Table 1:** Characteristics of total 116 patients who underwent glenoid lateralization design RSA (bony increased offset-reverse total shoulder arthroplasty, BIO-RSA) and humerus lateralization design RSA (curved short-humeral stem reverse total shoulder arthroplasty, ASCEND)

**Characteristic**	Glenoid lateralization RSA (**BIO-RSA, *n* = 60)**	Humeral lateralization RSA **(ASCEND, *n* = 64)**	***p*-value**
	**Mean ± SD**	**Mean ± SD**	
Sex (M/F)	15/45	14/50	0.682
Age (yr)	73.2 ± 5.3 (64–87)	75.0 ± 7.2 (56–88)	0.134
BMI	25.2 ± 4.5 (16–39)	24.9 ± 3.2(19–3)	0.691
Glenoid type (Walch et al.)	A1:34, A2:7, B1:13, B2:4 B3:1, C:1	A1:31, A2:16, B1:14, B2:2 B3:1	0.814
Follow-up (months)	38.5 ± 21.5 (18–86)	34.4 ± 13.1( 18–72)	0.209
Affected arm (Rt/Lt)	45/15	26/38	0.066

In our study, we used the glenoid and humeral lateralization designs consecutively because the humeral lateralized design was introduced in Korea later than the glenoid lateralized design. In the early phase of this study, we used the BIO-RSA or and then we used the Ascend Flex design, consecutively. All patients were followed for mean 3 years.

In Group I, cemented stem were used, and in Group II, cementless stem were used. Group I (glenoid lateralization design) included the use of (a) an autologous, trapezoidal bone graft harvested from the humeral head to restore the glenoid bone deficiency and lateralize the COR of the RSA (BIO-RSA, Tornier, Inc., Edina, MN) [11]. In Group I, the humeral component had an NSA of 155º with 20 º retroversion. Compared to Group I, in Group II, a curved, short humeral stem (Aequalis Ascend Flex, Wright Medical, Memphis, TN) was used. The humeral component had an NSA of 132º with 20º retroversion, a curved, hydroxyapatite-coated short-stem with an eccentric reverse tray (+ 1.5 mm and + 3.5 mm), and an asymmetric polyethylene insert (thickness + 6 mm and + 9 mm) with 12.5° of inclination.

The functional and radiologic outcomes were assessed and compared between the 2 groups at the last follow-up. Clinical scores, such as ASES score (American Shoulder and Elbow Surgeons Shoulder Score), UCLA score (University of California at Los Angeles Shoulder Score), SST score (Simple Shoulder Test score), VAS (visual analogue score) and ROM (forward flexion, abduction, external rotation, and internal rotation) were also compared between the groups. Preoperative imaging included plain radiographs (anterior–posterior X-ray, Y lateral, and axillary views), computed tomography scans for glenoid bone deformity, and magnetic resonance images (MRI) to assess the status of the rotator cuff. On sagittal MRI, fatty degeneration and muscle atrophy were classified according to the Goutallier classification, and then the RSA was planned [19].

**Fig. 2:**
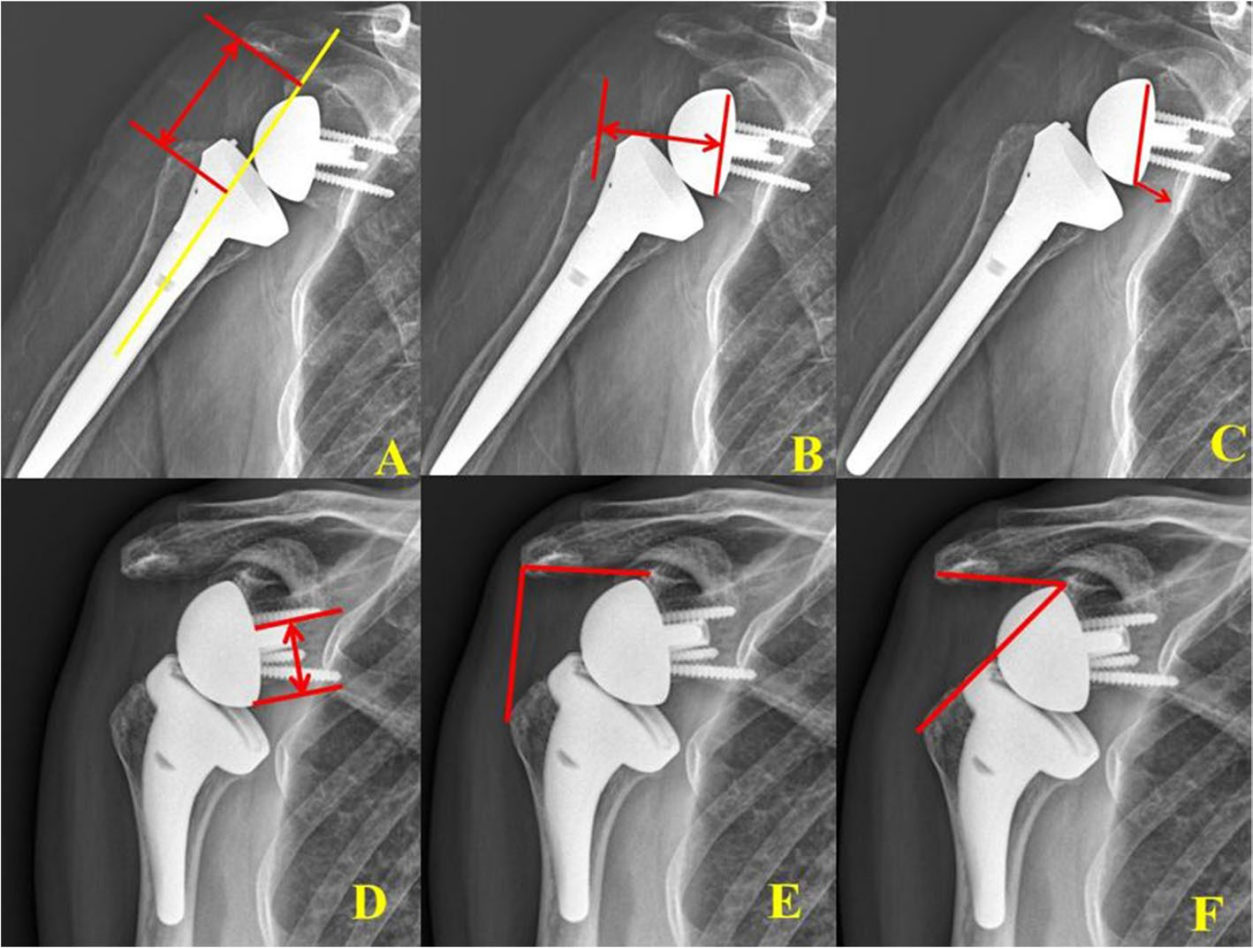
On the radiograph, we assessed several radiologic parameters and compared these between humeral and glenoid lateralization reverse total shoulder arthroplasty. **A** acromion-tuberosity (AT) distance, (**B**) glenoid-greater tuberosity (GT) distance*,* (**C**) prosthesis-scapular neck angle (PSNA) [20], (**D**) peg-glenoid rim distance (PGRD) [20], (**E**) lateralized shoulder angle, (**F**) distalization shoulder angle (DSA) [21]

Postoperative radiographs were taken in the immediate postoperative period and then at 3 months, 12 months, 24 months, and the last follow-up. On each radiograph, we assessed scapular notching, acromion-tuberosity (AT) distance, glenoid-greater tuberosity (GT) distance*,* prosthesis-scapular neck angle (PSNA) [20], peg-glenoid rim distance (PGRD) [20], scapular neck-inferior rim distance [2], lateralized shoulder angle (LSA), distalization shoulder angle (DSA) [21], bone graft incorporation, and loosening of the glenoid component and humeral stem (Fig. 2). GT distance and DSA means the amount of medialization in RSA. AT distance and DSA means the degree of humeral head lengthening. The severity of scapular notching was graded according to the Nerot-Sirveaux classification [22]. We used separated authors to check for preoperative radiologic finding (Lee HH) and outpatient score review (Park SE, Jun HS).

This research project has been reviewed and approved by the Institutional Review Board (IRB) of the authors’ affiliated institutions.

#### Surgical technique

All surgeries were performed by one senior author at a single center using the standard deltopectoral approach. Patients were placed under general anesthesia combined with an interscalene block and arranged in the beach chair position. After making a 10 cm longitudinal incision, the deltopectoral groove was found, and partial release of the pectoralis tendon insertion was done. The next step was identification of the biceps tendon in the bicipital groove, and then the biceps tendon was tenodesed in the upper bicipital groove. The subscapularis tendon was identified and detached from the lesser tuberosity. After the humeral head was dislocated, humeral head cut was made with an NSA of 155º with 20º retroversion in Group I. In group II, humeral head cut was made with an NSA of 135º with 20º retroversion.

During the humeral head cut in the BIO-RSA procedure, a 7 mm thick humeral head autograft was harvested in all patients because the 10 mm graft develops higher periarticular soft tissue tension and caused difficult prosthesis reduction in our patients. This graft was used to correct the glenoid deformity or glenoid bone defect (retroversion and inclination). After resurfacing the harvested bone according to the need for glenoid version and inclination, this trapezoidal autograft was attached to the long post baseplate (25 mm post length) of the glenoid component. This 25 mm baseplate was implanted over the glenoid’s inferior edge, and then a 36 mm glenosphere was placed over it. In our study, a cemented or cementless humeral stem was used in the BIO-RSA procedure. Sometimes, cortical breakage or cracking developed in the proximal humerus, and then cerclage wiring was used. In the humeral lateralization design, if the curved short-stem was used (Ascend flex, Tonier), routine glenoid preparation and glenosphere insertion were performed. Cancellous chip bone extracted from the resected humeral head was used for bone graft impaction of the proximal humerus. An onlay, curved short-stem was implanted after humeral canal reaming, and then the eccentric reverse tray was applied. After confirming the stability of the prosthesis, a polyethylene insert with 12.5º of inclination was implanted over the humeral stem, and then prosthesis reduction was performed.

Postoperatively, the arm was placed in an abduction brace for 4 weeks, but pendulum exercise was allowed immediately after the operation. After 4 weeks, the abduction brace was removed and then active assisted ROM exercise was initiated progressively. At 12 postoperative weeks, patients were encouraged to perform strengthening exercises and daily activities as tolerated.

### Statistical analysis

Continuous variables are reported as means ± standard deviations. Discrete variables are reported as numbers and percent of the total. Pearson’s chi-squared tests were used to compare binary variables (demographic data and complications). Differences between the two groups were calculated using the independent samples Student t-test for continuous measures and scores and the chi-square test for categorical measures.

The sample size was estimated using an effect size of 0.5, an acceptable alpha error of 0.05, and a beta error of 0.2 to ensure a power of 80%. The calculation was made to determine whether a sufficient number of samples had been collected for comparison. The adequate sample size was estimated to be 124 shoulders. In our study, 124 shoulders of 122 consecutive patients were included and analyzed (Group I: 60 shoulders, Group II: 64 shoulders). The statistical software used for all analyses was SPSS 22.0 (SPSS Inc., Chicago, IL, USA). *P*-values < 0.05 were considered significant.

## Results

### Clinical outcomes

The mean age of the patients was 73.7 ± 6.4 (64–85) years for Group I, and 74.0 ± 7.3 (56–91) years for Group II. The mean follow-up periods for Groups I and II were 39 months (18–86 months) and 34 months (18–72 months), respectively. The causes for RSA were rotator cuff tear arthropathy (55% of Group I and 61% of Group II), massive cuff tear (33% of Group I, 32% of Group II), and osteoarthritis (12% of Group I, 7% of Group II). Pre-operative and post-operative ASES, UCLA, SST, VAS, and ROM were evaluated and compared between the two groups (Tables 2). Both groups showed statistically significant improvements in clinical outcomes (*p* < 0.05). The two groups did not differ significantly in any of the clinical outcomes or ROM measures (*p* > 0.05) (Table 2).

**Table 2:** Comparison of clinical outcomes between glenoid and humerus lateralized design of reverse shoulder arthroplasty

Clinical outcomes	Glenoid lateralization RSA(BIO-(BIO-RSA, *n* = 60)	Humeral lateralization RSA (ASCEND, *n* = 64)	*p*-value
	**Mean ± SD**	**Mean ± SD**	
VAS	Pre / Post-operative 5.0 ± 1.6 / 2.6 ± 1.5	Pre / Post-operative 5.1 ± 1.7 / 2.6 ± 2.1	0.283 / 0.941
ASES	41.8 ± 23.1 / 69.2 ± 17.6	46.6 ± 24.5 / 69.3 ± 15.8	0.519 / 0.992
UCLA	17.7 ± 8.5/ 27.3 ± 6.1	18.9 ± 6.7 / 28.0 ± 3.8	0.519 / 0.540
SST	4.9 + -4.0 / 7.8 ± 1.9	5.2 + -2.0 / 8.6 ± 2.6	0.664 / 0.105
Preoperative ROM			
Forward flexion (°)	103 ± 36 / 134 ± 18	109 ± 41 / 139 ± 16	0.536 / 0.218
Abduction (°)	94 ± 38 / 133 ± 19	104 ± 39 / 138 ± 16	0.253 / 0.157
External rotation at side (°)	22 ± 13 / 25 ± 12	24 ± 11 / 29 ± 12	0.470 / 0.055
Internal rotation at back	L3 / L1	L3 / L2	0.933 / 0.512

### Radiological outcomes

Group I (glenoid lateralization group) showed bone graft incorporation in all patients, with no evidence of graft resorption. It did not differ significantly from Group II (humeral lateralization group) in scapular notching (*p* = 0.774), PSNA, PGRD, or AT distance. However, GT distance (*p* < 0.001) and DSA (*p* = 0.035) did differ significantly between the groups (Table 3). In Group II, larger GT distance and less DSA were found. Compared with Group I, Group II typically showed cortical bone thinning or erosion (absorption) between the humeral stem and lateral humeral cortex (Fig. 4A). However, no functional impairment or revision surgery occurred due to cortical thinning or erosion at last follow-up.

**Table 3:** Comparison of radiographic outcomes between glenoid and humerus lateralized design of reverse shoulder arthroplasty

**Characteristic**	Glenoid lateralization RSA (**BIO-RSA, *n* = 60)**	Humeral lateralization RSA **(ASCEND, *n* = 64)**	***p*-value**
	**Mean ± SD**	**Mean ± SD**	
PSNA^a^	106.1 ± 22.1 (77.0–144.0)	108.1 ± 18.7 (79.8–169.8)	0.611
PGRD ^b^	23.2 ± 9.6 (13.4–60.5)	24.0 ± 3.9 (17.8–36.0)	0.746
Scapular neck-inferior rim distance	2.9 ± 1.8 (-1.5–8.5)	2.5 ± 2.0 (-2.8–10.5)	0.771
AT distance ^c^	42.7 ± 10.7 (25.8–64.0)	46.3 ± 9.8 (23.6–65.6)	0.069
GT distance^d^	43.8 ± 9.9 (28.1–64.7)	51.6 ± 5.9 (36.8-.68.4)	0.000
Scapular notching			0.134
No notching	17	20	
Grade 1	18	30	
Grade 2	20	10	
Grade 3	4	1	
Grade 4	1	3	
LSA^e^	90.5 ± 9.2 (76.2–118.7)	89.2 ± 9.2 (69.1–123.2)	0.459
DSA^f^	80.4 ± 8.9 (61.0–113.9)	76.9 ± 8.3 (60.9–100.1)	0.035

*PSNA*^a^ *prosthesis-scapular neck angle*, *PGRD *^b^ *peg-glenoid rim distance*, *AT*^c^* distance* acromion-tuberosity distance, *GT*^d^*distance* Glenoid-greater tuberosity distance, *LSA*^e^ lateralization shoulder angle, *DSA*^f^ Distalization shoulder angle

### Complications

**Fig. 3:**
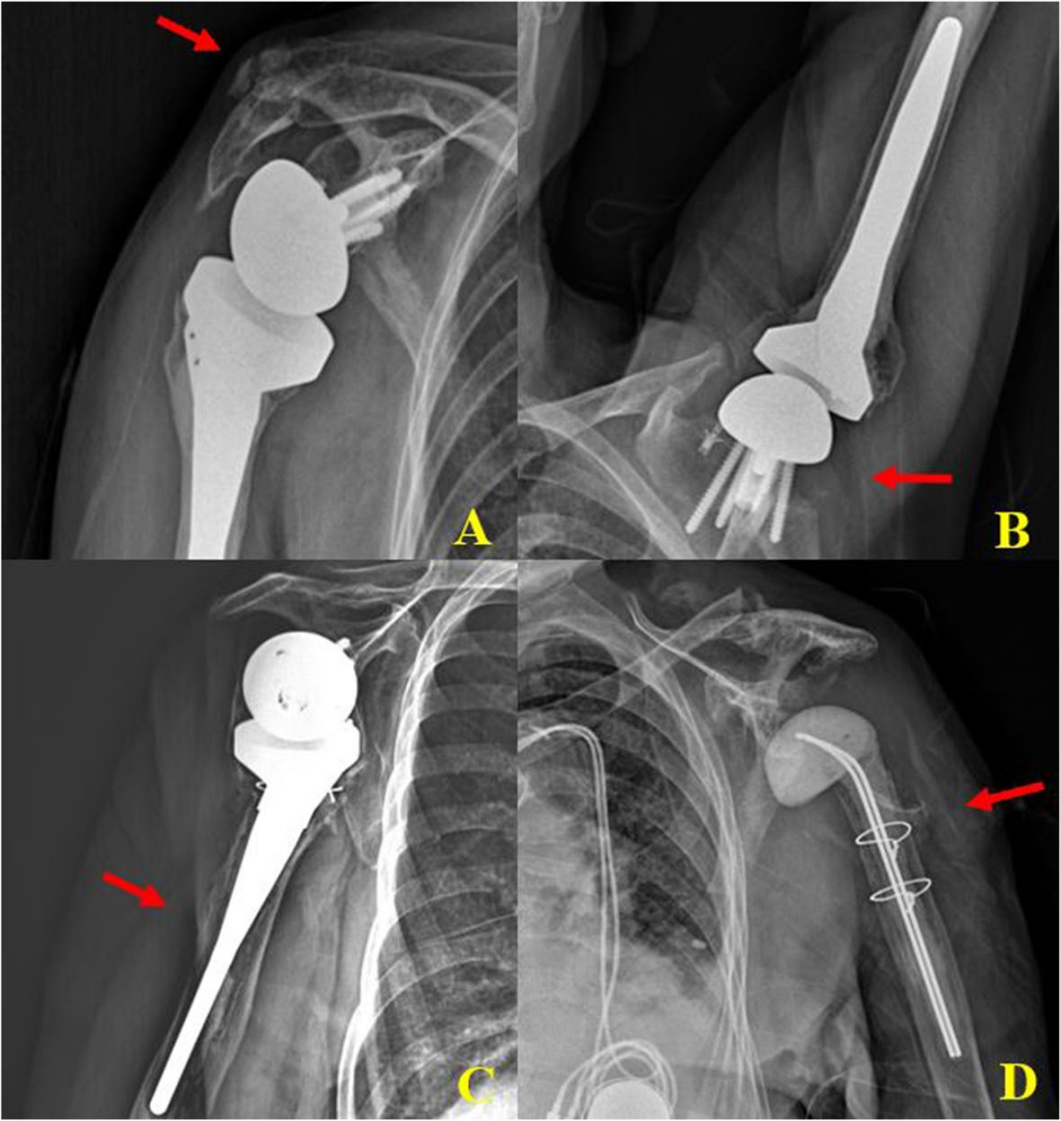
Radiologic complications of glenoid lateralization reverse total shoulder arthroplasty. Acromion fracture with scapular neck fracture (**A**). Axillary lateral view showing Grade 4 scapular notching (**B**). Humeral stem loosening was found in a 5-year follow-up X-ray (**C**). PROSTALAC insertion after implant removal was performed in a late infection (postoperative 3 years) (**D**)

Between 2 groups, there was no significant difference in the complication (Table 4). In Group I, one patient had humeral stem loosening, but she refused revision surgery due to her old age (86 years). One patient with PJI infection, had implant removal, debridement, and PROSTALAC insertion was done. One peri-prosthetic humerus shaft fracture was stabilized with cerclage wire. Two patients suffered a scapular neck fracture and acromion fracture (type II, fractures of the acromion posterior to the acromioclavicular joint) that was managed conservatively (Fig. 3).

**Table 4:** Perioperative complications between two different design of reverse total shoulder arthroplasty

**Complication**	Glenoid lateralization RSA (**BIO-RSA, *n* = 60)**	Humeral lateralization RSA **(ASCEND, *n* = 64)**	***p*-value**
Loosening	1	4	0.197
Humeral stem	1	3	
Glenosphere	0	1	
Periprosthetic humerus fracture	1	0	0.302
Infection	1	0	0.302
Periscapular fracture	1 (scapular neck) 1 (acromion)	1 (scapular neck) 1 (acromion)	0.948
Total		6	0.839

**Fig. 4:**
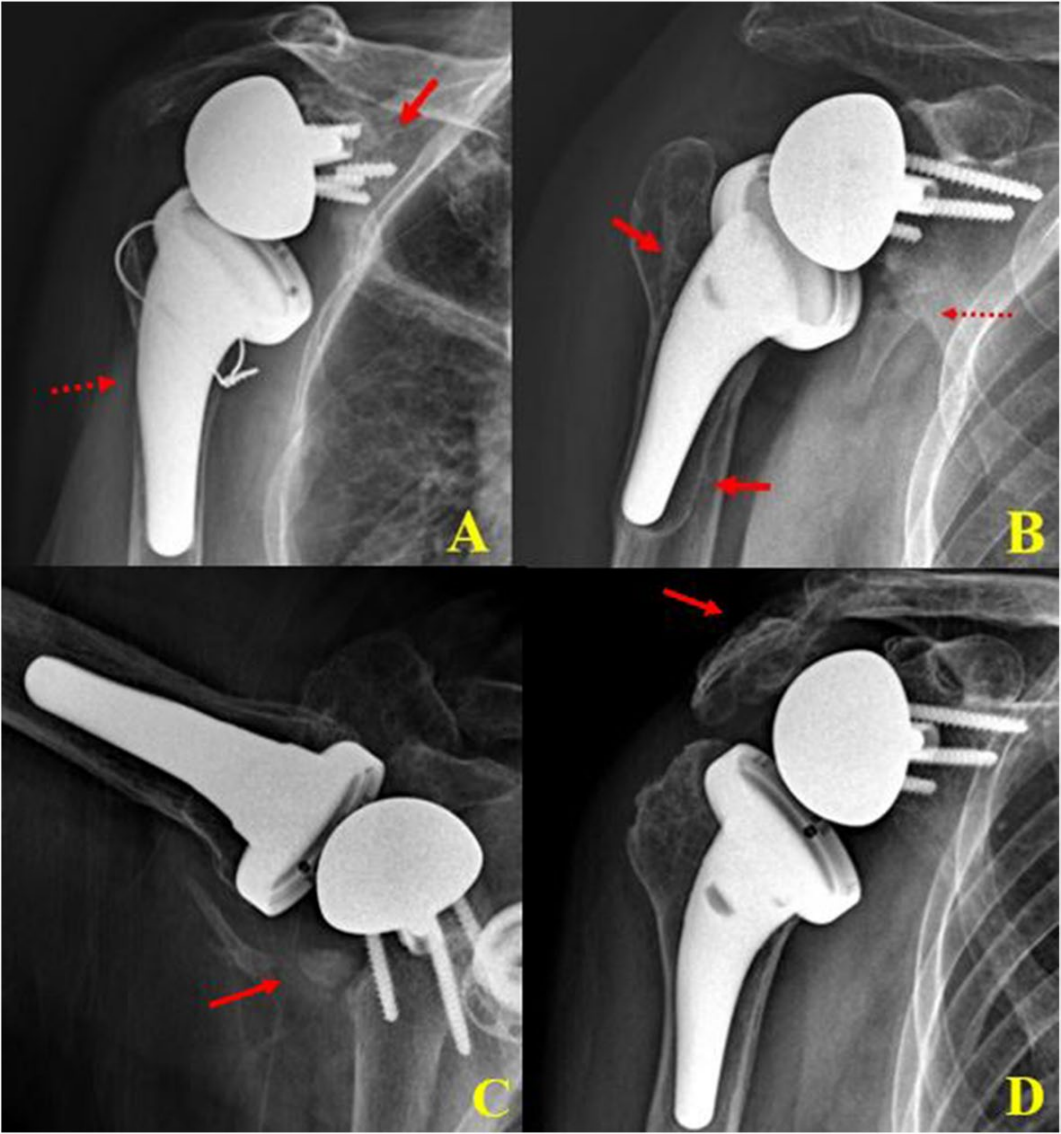
Radiologic complications of humeral lateralization reverse total shoulder arthroplasty. Glenoid loosening with broken screw (arrow) and high adaptation of the humeral stem (dotted arrow) (**A**). Scapular neck fracture (dotted arrow) and trauma-related humeral stem loosening (arrow) (**B**). Axillary lateral view showing Grade 4 scapular notching (**C**). Humeral stem loosening was developed in a 5-year follow-up X-ray (**C**). Late acromion fracture was developed and conservative treatment was done (**D**)

**Fig. 5:**
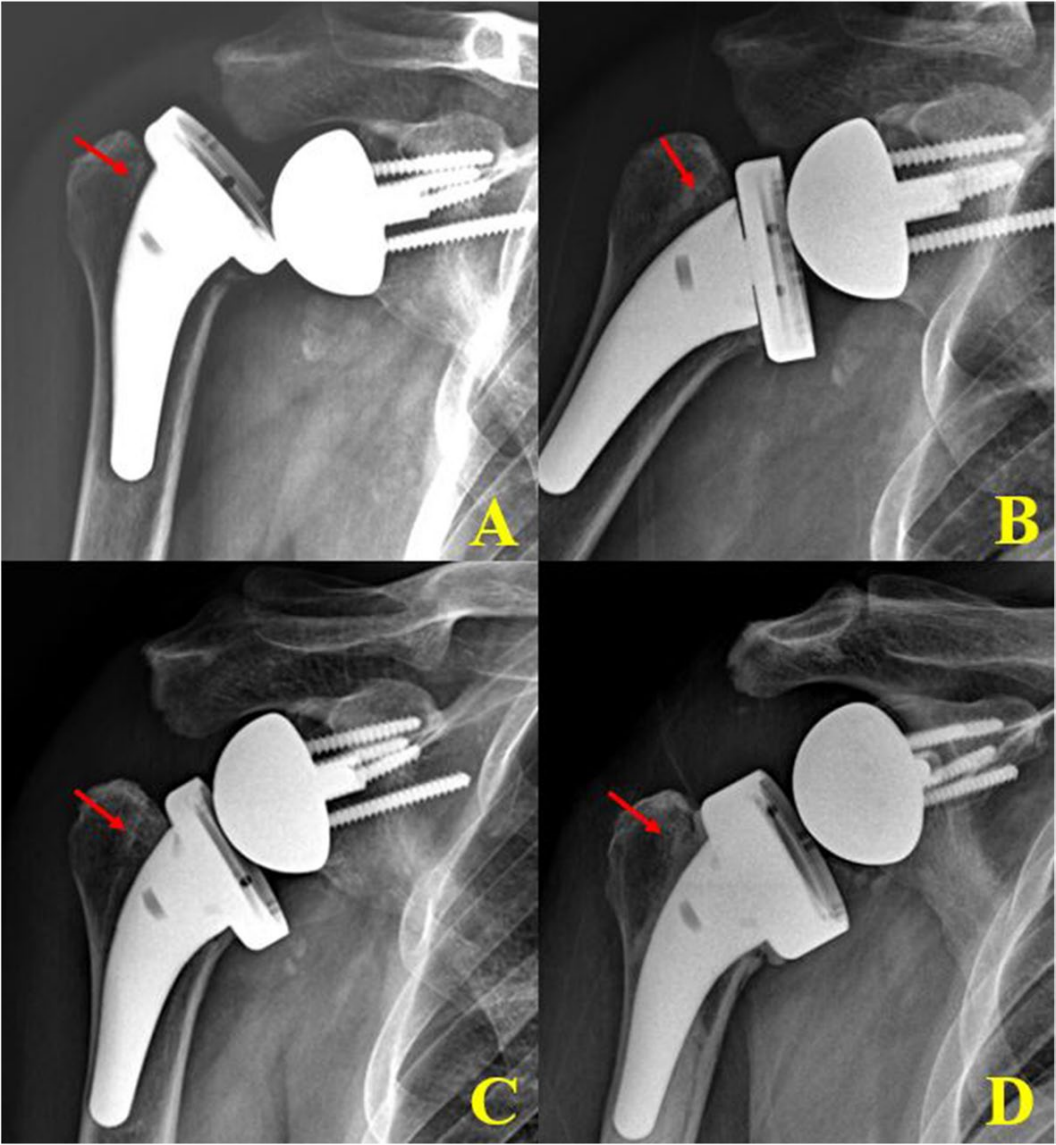
In a 65-year-old male patient with chronic alcoholism and recurrent shoulder dislocation, the first-time shoulder dislocation caused no loosening of the humeral stem (**A**). The second shoulder dislocation caused definite loosening of the humeral stem (**B**). Recurrent shoulder dislocation caused further stem loosening (definite large gap between humerus and stem) (**C**). With easy removal of the non-cemented humeral stem, revision surgery using a cemented humeral stem was performed (**D**)

In Group II, one patient had acromion fracture (type II fracture) and one scapular neck fracture that were managed conservatively. Four episodes of loosening (1 glenoid and 2 humeral stem) occurred (Fig. 4). Among these loosening (1 glenoid, 3 humerus), 2 revision surgery of humeral stem loosening was performed, and the other 2 patients refused the revision surgery due to their old age (age 85 years). During the revision surgeries, the loosened humeral stem was removed and then revision with cement humeral stem was performed (Fig. 4). One patient with chronic alcoholism had recurrent shoulder dislocation (more than 5 times), and then stem loosening developed (Fig. 5).

## Discussion

In our study, both groups showed no statistically significant differences in clinical and radiological outcomes, especially scapular notching between two glenoid and humeral lateralization designs at mid-term follow up (mean 3 years). However, humeral lateralization design showed larger GT distance and less DSA. Humeral lateralization design RSA could preserve the normal shoulder contour due to a larger GT distance (more lateralization) and provide less deltoid tension due to less DSA (less distalization of COR).

Compared with conventional RSA, the rates of scapular notching with humeral lateralization RSA was already known to be lower [4, 5]. Also, several authors reported decreased scapular notching and improvement in rotation with glenoid lateralization [10, 13, 23]. However, few studies have compared the glenoid versus humeral lateralized RSA designs, especially in an Asian population.

Scapular notching appears between 6 and 14 months postoperatively with an incidence of 44% to 96% [14]. and notching has been found to be associated with worse patient reported outcomes, function, and a higher complication and revision[24, 25]. Modern RSA designs combine a curved, onlay humeral component with a large, lateralized glenosphere placed in 10° of inferior tilt with > 3.5 mm of inferior overhang have been found to provide excellent results [26]. Only a few biomechanical studies have analyzed the effects of both humeral and glenoid lateralization [7, 27]. Biomechanical studies have shown higher ROM with decreasing NSA. A lower NSA raises the ROM before contact with the scapular pillar takes place and lowers the contact area at the inferior margin of the scapula, indicating that less scapular notching would be a likely consequence. Lateralization could provide restoration of the length of the horizontal rotator cuff muscle tendon units (cuff equilibrium) and lengthening of the moment arm of the deltoid muscle. In a simulation study, Ji et al. [28] demonstrated that an NSA of 135° produced the maximum total horizontal ROM at both 30° and 60° of scaption, regardless of the retroversion angle, and reduced the chances of scapular impingement. Werner et al. [8] used a computer model to show that combining a 135° model with 5 mm of glenoid lateralization provided the best results in impingement-free ROM, except for abduction.

With both the glenoid and humeral lateralized designs, the aim of lateralization was met, but the clinical outcomes did not differ significantly from those with conventional RSA design, except external rotation [18]. In a systematic review of the post-operative RSA outcomes between medialized versus lateralized glenoid implants. They were able to show significantly better external rotation (mean 21◦ vs. 7◦) in the glenoid lateralization, but there were no significant differences in clinical scores [29].

By lateralizing the COR, both lateralization designs could reduce the workload on the deltoid to allow the maintenance of active external rotation even in the absence of the teres minor [30]. Athwal et al.[9] concluded that the rate of scapular notching was significantly higher in the standard RSA group than the BIO-RSA group, but no other outcome measures differed statistically, including ROM, strength, and validated outcome scores. Merolla et al. [5] reported 5% notching in patients with Ascend Flex group (humeral lateralized design), compared to 39% notching in patients with Aequalis II group (conventional design). Although scapular spine fractures occurred only in the Ascend Flex group, the difference between the 2 groups was not significant. Boileau et al. [11] reported mild notching in 25% patients with glenoid lateralization RSA using a humeral autograft (BIO-RSA). A recent systematic review (> 6000 RSA from > 100 studies) showed no statistical significance in mechanical loosening between lateralized and medialized RSA (1.15% medialized vs. 1.84% lateralized) [31]. In our study, both the glenoid lateralized (BIO-RSA) and humerus lateralized (curved, short-stem) groups showed no significant differences of scapular notching (*p*-value = 0.134, Table 3). East Asian population has a smaller glenoid than the populations of North American in previous studies, especially the females [32]. However, different design RSA didn’t show the difference of scapular notching on the smaller glenoid size of Asian[33].

All the functional scores were similar between the groups with a mean 3-years follow up. There were no significant differences in the improvement of forward flexion, abduction, external rotation, or internal rotation between two groups. In the radiologic outcomes, AT distance, PSNA, PGRD, and the scapular neck-inferior rim distance did not differ significantly. However, 2 radiologic parameters including larger GT distance and decreased DSA was different. Thus, the humeral lateralized group could preserve the normal shoulder contour and anatomy better than in the glenoid lateralized group [5]. Also, there were no significant differences of the complications between 2 groups. The short-stem allows easier anatomic revision of the inverse prosthesis without stem removal [5]. In the conventional Grammont design, stem fixation is diaphyseal, so it develops stress and could increase the risk of late periprosthetic fractures [34]. Proximal and humeral shaft fractures are common in elderly people due to osteoporosis, implant-related stress riser development, and low energy trauma. Such periprosthetic humeral fractures in diaphyseal stem prostheses have to be revised in most cases and negatively affect the results [35]. Ehud et al. [36] reported a low rate of complications with a short humeral stem, and the most frequent complication was late traumatic periprosthetic metaphyseal fractures, which were mainly treated conservatively. Merolla et al. [5] concluded that patients with humeral lateralization using an onlay, curved short-stem have a higher scapular fracture rate than those treated with the conventional Grammont design (statistically not significant) but lower rates of scapular notching (*p* = 0.0003). However, our study showed no significant differences of scapular and acromion fracture between 2 groups. Compared to the glenoid lateralization (BIO-RSA), which could increase shear forces on the interface between the glenoid and the baseplate [37], humerus lateralization may have lower loosening rates of glenoid component. However, there was no higher loosening rate of the glenoid component of BIO-RSA in our study. In humeral lateralized group, 2 revision surgery of the traumatic loosening of a non-cemented humeral stem was performed. A multicenter study by Haidamous et al. [38] showed that distalization had a significantly higher incidence of acromial fractures and they did not detect a negative influence of lateralization. Put it all together, our study showed that humeral lateralization design showed a statistically significant difference in larger GT distance and decreased DSA, in which meant that humeral lateralization design showed positive effect of lateralization (larger GT distance) without a negative influence of distalization (decreased DSA).

Typically, compared with the glenoid lateralized design, the humeral lateralized design (curved, short stem) showed only specific radiologic findings, such as cortical thinning of the lateral humeral cortex or cortical erosion due to contact between the humeral stem and the lateral humeral cortex. However, those cortical bony erosion didn’t cause any functional disability or require revision surgery of the humeral stem at mid-term follow-up. A previous study showed that radiologic changes are associated with a higher filling ratio and cortical contact of the stem [6].

This study had several limitations. First, this was a retrospective review of the consecutive case series, and patient assessments weren’t performed in the same time period. The curved short-stem (Ascend design) and BIO-RSA procedures were introduced in Korea at different time periods. Even though both groups had a minimum of 2 years of follow-up, which met the minimum qualification, longer term follow-up evaluation are needed. Second, we evaluated only 60 BIO-RSA (glenoid lateralization design). In our study, the adequate sample size in each group was estimated to be 64 patients (124 shoulders) and we enrolled 60 glenoid lateralization and 64 humeral lateralization group in this study. When trying to compare averages with the independent sample Student t-test, the minimun sample size was 64, which was a little short, but the study was started thinking that it was an approximation. Even though adequate sample size (64 patients) was not reached in group I (60 patients), the difference is not so great and more patients should be included in the future study. Third, we only used cementless short-stems in the humeral lateralized RSA. In the glenoid lateralized RA, we used cemented long stem. Because curved short-stems without cement could be more susceptible to trauma, cemented short-stems might show different outcomes for humeral stem loosening in longer term follow-up and different designs of humeral lateralization group, such as a straight stem should be considered in a future comparative study. Furthermore, the subjective measurement deviation in the parameters of Fig. 2 might be large and trained 2 fellows tried to reduce these subjective measurement deviation.

## Conclusions

The clinical and radiologic outcomes of the two groups did not differ significantly, including scapular notching at mid-term follow-up. However, humeral lateralization design showed larger GT distance and less DSA. Humeral lateralization design RSA could preserve the normal shoulder contour due to a larger GT distance (more lateralization) and provide less deltoid tension due to less DSA (less distalization of COR).

## Data Availability

The datasets generated and/or analysed during the current study are not publicly available due to consent of publication authority but are available from the corresponding author on reasonable request.
